# Knowledge, Attitudes and Perceived Barriers to Pneumococcal Vaccination: A Cross-Sectional Survey Among Healthcare Workers and Administrative Staff at an Italian University Hospital

**DOI:** 10.3390/vaccines14060530

**Published:** 2026-06-15

**Authors:** Giulia Congedo, Rossella Mancini, Fabio Pattavina, Domenico Pascucci, Stefania Bruno, Patrizia Laurenti

**Affiliations:** 1 Department of Life Sciences and Public Health, Università Cattolica del Sacro Cuore, 00168 Rome, Italy; giulia.congedo01@icatt.it (G.C.); rossella.mancini01@icatt.it (R.M.); domenico.pascucci@policlinicogemelli.it (D.P.); stefania.bruno@unicatt.it (S.B.); patrizia.laurenti@unicatt.it (P.L.); 2 Fondazione Policlinico Universitario A. Gemelli IRCCS, 00168 Rome, Italy

**Keywords:** pneumococcal vaccination, healthcare workers, knowledge, attitudes, vaccine coverage

## Abstract

**Introduction**: *Streptococcus pneumoniae* severely affects adults over 65, especially those with comorbidities. Since vaccination coverage among healthcare workers (HCWs) is unknown despite free availability, this study evaluates knowledge, behaviours, hesitancy and accessibility among employees of an Italian hospital. **Methods**: A prospective cross-sectional survey was administered via “SurveyMonkey.” From February 22 to June 15, 2024, healthcare and administrative staff aged ≥ 18 at the Fondazione Policlinico Universitario Gemelli were recruited by email. Descriptive and inferential analyses used Stata 16.1. **Results**: Among HCWs, 72% are women, with an average age of 48. Pneumococcal vaccination coverage is 20%, with 82.7% vaccinated in-hospital. Preferred information sources include courses, webinars, and institutional websites. For management staff, vaccine safety and effectiveness were significant determinants. Among administrative employees, 65% are women (average age 51); 19% are vaccinated, 24% are unsure, and 43% prefer in-hospital vaccination. Physicians cited trust in vaccines (25.3%) and self-protection (23.2%) as key motivators, compared with 12.4% among nursing, technical and rehabilitative staff. Recommendation to family members was higher among medical and specialist professionals (90%) than in other groups (77% in nursing/technical/rehabilitative; <50% in assistants and auxiliary staff). About half of the groups rated their knowledge at level 2 (scale 1–4). Multivariable regression analysis showed that medical professionals and specialists exhibited a higher perception of the importance and safety of vaccines compared with other categories. **Conclusions**: HCWs showed greater knowledge of pneumococcal vaccination, while administrative staff had lower awareness and more hesitancy. Both groups preferred in-hospital vaccination and expressed interest in structured educational initiatives.

## 1. Introduction

*Streptococcus pneumoniae* primarily affects the most vulnerable age groups, particularly new-born children, and adults over 65 [1,2]. Advanced age, pre-existing chronic health conditions such as lung diseases, heart disease, diabetes mellitus, and hematologic diseases, significantly increase the likelihood of severe pneumococcal infections and related complications [3]. For this reason, pneumococcal vaccination is included among the recommended vaccinations for certain at-risk categories in the 2023–2025 National Vaccine Prevention Plan (PNPV) [4]. Vaccination helps reduce the risk of infections, prevents complications, and decreases hospitalisation and mortality rates. In 2023, two types of pneumococcal vaccines were offered for free to the above mentioned categories: the Pneumococcal Conjugate Vaccine (PCV 13), intended for patients of any age at risk due to chronic conditions, as well as for patients aged ≥ 65 years, in both cases without prior vaccination, and the Pneumococcal Polysaccharide Vaccine (PPV23), recommended for patients vaccinated with PCV 13 who had not yet been vaccinated with PPV23 [4]. According to the Italian National Institute of Health, invasive pneumococcal disease cases declined by about one-third in 2020 compared to the previous two years. However, this result may have been impacted by the containment measures implemented during the COVID-19 pandemic and a subsequent lack of available data [3]. In 2022, an increase in the incidence of invasive pneumococcal diseases was observed compared to 2021. In adults over 64 years old, the incidence rose to 4.39 cases per 100,000 inhabitants in 2022, which is double the value for 2021. Additionally, among adults over 64 years, the percentage of cases caused by serotypes included in the vaccines gradually increased over the three-year period from 2020 to 2022, reaching 39% (162 of 416 cases) for the 13-valent vaccine and 70% (293 of 416 cases) for the 23-valent vaccine in the last year [5]. Nevertheless, there is still a lack of a planned and uniform monitoring system for vaccination coverage across Italy which, according to the new 2023–2025 PNPV, should be at least 75% [4]. The 2023–2025 PNPV on vaccination coverage data for the general population does not include data on healthcare workers (HCWs), considered a category at risk due to their contact with patients and for which vaccination is recommended and offered for free. The offered schedule (one dose annually, starting at least from the cohort of individuals aged 65 and over) should be supplemented with a sequential schedule (PCV + PPV) depending on the type of PCV vaccine used [4,6]. Since the purpose of vaccination is threefold—protecting the worker, protecting at-risk patients the worker may come into contact with, and preventing the interruption of essential healthcare services—each healthcare institution needs to actively promote all initiatives aimed at increasing vaccination uptake among their staff, considering the evidence of risk reduction [7,8]. While vaccine hesitancy among HCWs has been extensively studied in the context of influenza and, more recently, COVID-19 vaccination, comparatively little attention has been paid to pneumococcal vaccination attitudes and coverage within hospital workforces, particularly in the Italian context. Furthermore, most existing surveys focus exclusively on clinical staff, leaving administrative and auxiliary personnel—who represent a sizeable proportion of hospital employees and interact regularly with at-risk patients—largely unexamined. Against this background, the present cross-sectional study aimed to investigate knowledge, attitudes and behaviours regarding pneumococcal vaccination among both healthcare and administrative staff at a large Italian university hospital. Secondary objectives were to assess vaccine hesitancy prevalence across professional categories and to describe vaccine accessibility preferences, in order to inform targeted institutional interventions.

## 2. Methods

### 2.1. Study Design

This study is a cross-sectional study carried out through an online survey administered via SurveyMonkey, with the questionnaire available from February to June 2024.

A non-probability, voluntary response sampling method was adopted. All employees of the Fondazione Policlinico Universitario Agostino Gemelli IRCCS were invited to participate in the study via an institutional email containing a link to the online questionnaire. Participation was entirely anonymous and voluntary. Respondents were categorised as healthcare workers—including doctors, nurses, and other healthcare workers—or administrative staff, such as accountants, logistics personnel, and various technical specialists. Only fully completed questionnaires were included.

A minimum sample size of 452 HCWs and 158 administrative staff was estimated a priori to investigate the study variables within the target healthcare worker population. Sample size calculation was performed using a standard sample size calculator based on the reference populations of the two professional groups, assuming a response distribution of 50%, a 95% confidence level, and a 5% margin of error.

#### Questionnaire and Data Collection

The questionnaire used in this study was purpose-built by the authors. Prior to being administered to the study participants, the questionnaire was preliminarily tested with approximately twenty members of the hospital’s administrative and clinical staff, to evaluate its comprehensibility, appropriateness, and overall effectiveness. The online survey featured 52 closed-ended questions across five sections, with two distinct versions designed to reflect the different levels of specific knowledge within the professional categories, one for healthcare workers and one for administrative staff. While both versions shared the same overall structure, the healthcare worker version included additional questions tailored to clinical practice, particularly focusing on pneumococcal disease, vaccine indications, and pathogen-specific knowledge.

**Section A:** Collected sociodemographic and work-related data (age, gender, marital status, education, occupation, workplace). This section aimed to analyse vaccine hesitancy and attitudes in relation to sociodemographic variables.

**Section B:** Examined health conditions, focusing on chronic diseases and factors increasing susceptibility to severe pneumococcal infections. This section assessed whether health conditions influence vaccine hesitancy or attitudes towards pneumococcal vaccination.

**Section C:** This section explored participants’ knowledge on pneumococcal vaccines, including perceptions of their safety, effectiveness, and views on pneumococcal disease. It played a key role in assessing overall attitudes and identifying factors associated with vaccine hesitancy.

**Section D:** Investigated vaccination-related intentions, behaviours and attitudes, including previous vaccinations, co-administration, and willingness to vaccinate others. This directly contributed to investigating vaccine accessibility, gathering data on vaccination behaviours and willingness to promote vaccination.

**Section E:** Focused on knowledge of pneumococcal vaccines, information sources, preparedness, topics for further exploration, and communication channels. This section identified knowledge gaps and assessed communication effectiveness, offering insights to improve vaccine literacy and accessibility for hospital staff.

Several sections of both questionnaires allowed multiple responses: for example, Section B (risk factors), Section C (disease outcomes), Section D (vaccination convenience and reasons for vaccination choices), and Section E (information channels). For administrative staff, Section E also included two additional multiple-choice questions on sources of information and topics of interest.

The questionnaire underwent pilot testing in a sample of 13 participants, including 8 healthcare workers and 5 administrative staff members, to assess item comprehensibility and internal consistency. Participants rated each item on a 7-point Likert scale ranging from 1 (“not meaningful at all”) to 7 (“very meaningful”). A mean score > 5 was predefined as the acceptability threshold. All items included in both questionnaires exceeded this threshold, supporting their clarity and comprehensibility. Internal consistency was evaluated using Cronbach’s alpha coefficient. The resulting alpha values were 0.77 for healthcare workers and 0.72 for administrative staff, indicating satisfactory reliability of the questionnaire.

### 2.2. Statistical Methods

For the statistical analysis, HCWs were divided into three groups:

(1) Medical and specialist healthcare professionals (medical doctors, biologists, pharmacists, etc.).
(2) Nursing, technical and rehabilitative healthcare professionals (nurses, physical therapists, dietitians, etc.).
(3) Healthcare assistants and auxiliary staff (nursing assistants).

The 4-point Likert scale in Section C was also used in the questionnaire to investigate general knowledge about vaccination and specific knowledge regarding pneumococcal vaccine. Likert-scale answers were coded as “1” if “strongly disagree” and as “4” if “strongly agree”. The answers “agree” and “disagree” were coded as “3” and “2” respectively. Analysis stratified by educational level was performed exclusively for administrative staff, and the corresponding findings are presented in the Results Section 3.

Descriptive statistics combining measures of dispersion and central tendency were used. For qualitative data, absolute frequencies and percentages were calculated. For quantitative data, the mean or median with interquartile range or standard deviation was calculated. Likert-scale responses were treated as ordinal variables. Associations between participants’ characteristics and questionnaire outcomes were assessed using ordinal logistic regression models, with the outcome variables entered according to their ordered response categories. To check the normality of the data, the Shapiro–Wilk and/or Kolmogorov–Smirnov test was used. The chi-square test and a logistic regression model accepting a significance level of 0.05 was used to make comparisons between groups. Scale responses were analysed as categorical variables. Logistic regression analyses were performed to evaluate the associations between the study variables and the outcomes of interest. The results are reported as odds ratios (ORs) with corresponding 95% confidence intervals (95% CIs) and *p*-values.

Prior to model estimation, multicollinearity among independent variables was assessed using variance inflation factors (VIFs). Model fit was evaluated using the Hosmer–Lemeshow goodness-of-fit test. Independence of observations was ensured by the study design, as each participant contributed a single response to the dataset. In addition, frequency distributions were examined to verify adequate cell counts for categorical variables included in the analyses.

No adjustment for multiple comparisons was applied, as the analyses were based on prespecified hypotheses and multivariable regression modelling. Statistical significance was set at *p* < 0.05. Analyses were performed with Stata 16.1 statistical software (single-user perpetual—serial number 301606337087).

### 2.3. Ethical Approval and Informed Consent

The study was approved by the Ethics Committee of the Fondazione Policlinico Universitario A. Gemelli—IRCCS (prot. no 0021609/21 ID 4104). It was conducted in accordance with Good Clinical Practice (GCP) standards and the principles of the Declaration of Helsinki. Data were processed in compliance with EU Regulation 2016/679 (GDPR), Legislative Decree 196/2003, and current Italian data protection legislation. Informed consent was obtained from all participants prior to questionnaire completion, and data were collected and analysed in anonymous and aggregated form.

## 3. Results

Out of 6244 initial invitations, 1671 were undeliverable. Of the 4573 successfully delivered, 1352 recipients responded to the questionnaire (response rate: 29.6%). Among them, 1090 completed the survey in full, corresponding to a completion rate of 80.6%.

### 3.1. Healthcare Workers

Among respondents, the majority were female (71.78%), with a median age of 48 years (IQR 38–56). Most participants held a postgraduate or university degree (80.07%) and were employed as nursing, technical, or rehabilitative healthcare professionals (64.72%). Over half were married (52.95%), 24.42% reported being smokers, and 71.53% declared having at least one chronic condition (Table 1). The most frequently reported were thyroid disorders, hypertension, arthritis, and osteoarthritis.

### 3.2. Administrative Staff

Among the 197 administrative staff who completed the survey, the majority were female (64.97%), with a median age of 48 years (IQR: 38–56). In terms of education, nearly half had completed high school (49.75%), while 25.89% held a university degree and 21.31% had postgraduate education. Most respondents were married (52.79%), and 73.10% were employed in back-office roles. Regarding health status, 28.44% reported having at least one chronic condition, and 23.86% were current smokers (Table 1).

Following the baseline characterisation of healthcare workers and administrative staff, the analysis addresses the core thematic areas investigated in the survey.

The subsequent sections examine these specific dimensions in detail:

1. Knowledge and attitudes on vaccination in general and pneumococcal vaccination.
2. Vaccination-related intentions, behaviours, and attitudes.
3. Preferred channels for vaccine information (Section E), which are evaluated in the following section to identify the most effective communication pathways for each professional category.

To comprehensively interpret these results, advanced statistical analyses and regression models were performed to identify the main predictors influencing knowledge, attitudes, and intentions across the overall sample.

Table 2 presents the findings related to knowledge and attitudes concerning vaccination in general, as well as specifically for pneumococcal vaccination. All items show statistically significant differences (*p* < 0.05) among the three categories of healthcare professionals examined, indicating marked variation in responses depending on professional role. For instance, 47.73% of healthcare assistants and auxiliary staff agreed that immunity acquired through infection is preferable to vaccination, in contrast to only 7.38% of medical and specialist healthcare professionals. Similarly, while more than half of healthcare assistant and auxiliary staff (54.55%) believed that vaccines frequently cause adverse effects, this opinion was shared by just 25.09% of medical and specialist healthcare professionals.

Table 3 reports the vaccination-related intentions, behaviours, and attitudes of healthcare workers, including history of influenza and pneumococcal vaccination, and perceived knowledge about pneumococcal vaccines. Statistically significant differences (*p* < 0.05) emerged, for all items, among the three professional categories examined. Pneumococcal vaccination coverage was more frequent among medical and specialist healthcare professionals (24.07%) than among the other groups. When asked to rate their knowledge of the pneumococcal vaccine on a 4-point scale, the majority in all categories rated their knowledge as low (scores 1 or 2), with healthcare assistant and auxiliary technical staff most frequently reporting “not at all” (40.48%).

Institutional websites emerged as the preferred channel for obtaining information on pneumococcal vaccination (39.8%), followed by continuing education courses offering credits (34.2%), webinars (19.3%), and podcasts (6.8%). No significant differences in communication channel preferences were identified among the HCW categories surveyed (*p* = 0.578) (Table S1).

Administrative Staff expressed a preference for obtaining more information through online events (25.93%), institutional websites (41.51%), in-person events (25.32%) and podcasts (7.74%). The results show non-statistically significant values among the various communication channel methods (Table S2).

Statistical analysis of the respondents’ answers, stratified by educational level, revealed a homogeneity of perspectives among most of the surveyed items. Indeed, no statistically significant differences were observed among the groups regarding the perception of vaccines as an essential tool (*p* = 0.065), their impact on the spread of infectious diseases (*p* = 0.213), the risk–benefit ratio between disease complications and adverse effects (*p* = 0.134), the preference for natural immunity over vaccination (*p* = 0.18), the perceived severity of pneumococcal disease (*p* = 0.604), and the safety of available vaccines (*p* = 0.156).

Conversely, educational attainment was found to be statistically significant in determining technical knowledge and awareness of advanced epidemiological mechanisms, specifically regarding the number of available vaccines (*p* = 0.009) and the role of vaccination in countering antibiotic resistance (*p* = 0.032) (Table S3).

### 3.3. Multivariable Logistic Analysis

Table 4 shows the results of a multivariable logistic regression analysis comparing the three professional categories among healthcare workers. For healthcare assistants and auxiliary technical staff, no statistically significant differences emerged when compared to nursing, technical, and rehabilitation staff (reference group). In contrast, medical and specialist healthcare professionals showed statistically significant differences in relation to three statements: “Vaccines are an essential tool for protecting individuals and communities” (*p* < 0.001), “The risk of complications from pneumococcal disease is greater than the risk of serious adverse effects from vaccines” (*p* = 0.001), and “The vaccines available against pneumococcus are safe” (*p* = 0.001). All other items did not show significant associations.

In Table 5, which compares all healthcare workers (reference group) and administrative staff, only one statement reached statistical significance: “The risk of complications from pneumococcal disease is higher than the risk of severe side effects from vaccines” (*p* = 0.002), with administrative staff less likely to agree with this statement.

## 4. Discussion

This study aimed to investigate knowledge, attitudes, and behaviours regarding pneumococcal vaccination among healthcare and administrative staff in a large Italian university hospital. The survey revealed a low pneumococcal vaccination uptake (20% among healthcare workers and 19% among administrative staff), along with disparities in vaccine-related knowledge and confidence across professional categories. Physicians demonstrated the highest levels of awareness and acceptance, whereas nursing, auxiliary, and administrative personnel reported lower knowledge and more hesitancy.

Given the cross-sectional and self-reported nature of the data, all associations reported should be interpreted as descriptive correlates rather than causal determinants of vaccination behaviour; the study is not designed to establish directionality or causality.

This study makes several contributions that distinguish it from existing literature on healthcare worker vaccination. While prior surveys have extensively examined attitudes toward influenza and COVID-19 vaccines among HCWs, evidence specifically addressing pneumococcal vaccination in hospital staff remains sparse, both in Italy and in the broader European context. Furthermore, the simultaneous inclusion of administrative and auxiliary personnel—alongside clinical staff—allows a comparison that is rarely performed in the literature and reveals marked disparities in knowledge and vaccine confidence across professional groups working in the same hospital. Finally, the Italian institutional setting provides original coverage data that can directly inform locally adapted vaccination strategies within a national framework that targets but does not systematically monitor vaccination rates among hospital workers.

Despite the widespread availability and strong recommendations for pneumococcal vaccination—particularly for older adults and those with chronic conditions [4,6]—the results demonstrate a disappointingly low uptake of the vaccine among healthcare workers and administrative staff. These data reinforce concerns previously raised in the literature regarding persistent vaccine hesitancy and suboptimal adult immunisation coverage, even in professional healthcare settings [9,10,11,12,13,14].

One of the most salient findings is the disparity in vaccine knowledge and acceptance across different job categories. Medical and specialist healthcare professionals exhibited the highest level of understanding and trust in the vaccine’s safety and effectiveness, in line with other studies conducted in European and North American settings that consistently associate higher medical education with increased vaccine literacy and acceptance [15,16,17,18]. Notably, 90% of physicians indicated willingness to recommend pneumococcal vaccination to family members, underscoring the potential for these professionals to act as peer influencers.

The heterogeneity in vaccine knowledge and acceptance across professional categories calls for role-specific educational strategies rather than a one-size-fits-all approach. For medical and specialist healthcare professionals—who demonstrated the highest levels of vaccine literacy and confidence—institutional efforts should focus on formalising their role as peer champions and vaccination advocates. For nursing, technical and rehabilitative staff, the integration of targeted pneumococcal vaccination modules within existing ECM (Continuing Medical Education)-accredited training pathways appears particularly promising, given this group’s expressed preference for structured, credit-bearing educational formats. For healthcare auxiliary and administrative personnel—who showed the lowest awareness and the most hesitancy—simplified on-site vaccination access points, proactive institutional recall systems and brief digital information modules delivered via institutional websites align most closely with both the knowledge gaps and the communication preferences identified in this study. Across all groups, the marked preference for in-hospital vaccination delivery supports the institutionalisation of on-site, opportunistic vaccination hubs as a key structural intervention.

In contrast, nursing, technical, rehabilitative professionals and auxiliary staff demonstrated markedly lower levels of knowledge and vaccine confidence. The low rate of agreement with key vaccine-related statements reflects a potentially underestimated gap in continuous professional training among non-physician staff. These gaps in knowledge mirror the findings of Tomboloni et al. (2019) [19] and Evren et al. (2020) [17], who reported a lack of structured educational interventions for nurses and hospital support staff, contributing to lower vaccination adherence that could lead to risk increasing [17,19].

Moreover, the administrative staff subgroup showed both the lowest awareness and the weakest perceptions of vaccine efficacy and safety. A significant proportion (24%) of respondents were unaware of their own vaccination status, suggesting the need for more robust occupational health documentation and proactive vaccination recall systems. These results are consistent with qualitative findings by Fuller et al. and Huang et al., which highlighted lack of information, unclear institutional communication, and procedural complexity as major hindering factors to adult vaccination [10,12].

Several mechanisms may help explain the lower knowledge and vaccine confidence observed among administrative and auxiliary staff, although these remain speculative in the absence of direct measurement. First, these groups typically have fewer interactions with occupational health services than clinical personnel, reducing both their exposure to vaccination recommendations and their opportunities for opportunistic vaccination. Second, their professional environment provides less routine engagement with health-related information, which may contribute to comparatively lower health literacy with respect to specific vaccine-preventable diseases such as pneumococcal infection. Third, institutional communication about vaccination schedules is often channelled through clinical hierarchies and professional medical networks, generating an information asymmetry that systematically disadvantages non-clinical employees. Finally, administrative staff may perceive their own occupational risk of pneumococcal disease as lower than that of clinical personnel, potentially attenuating their motivation to seek vaccination. These hypotheses warrant direct investigation in future research through qualitative or mixed-method approaches capable of capturing the lived experience of non-clinical hospital employees in relation to occupational health services.

Interestingly, preferences for receiving vaccines within the hospital setting were widespread, particularly among administrative and healthcare assistant and auxiliary staff. This preference—confirmed by the fact that 82.7% of flu-vaccinated respondents preferred receiving their vaccine on-site—underscores the potential of hospital-based vaccination hubs. On-site access facilitates convenience, minimises work disruption, and may reduce structural barriers to vaccination, especially among non-clinical staff who may have fewer interactions with occupational health services. These findings support existing evidence from both European and international contexts, which advocate the institutionalisation of on-site, opportunistic vaccination delivery models to boost coverage among HCWs [7,8,20].

Another key implication concerns the preferred communication channels for vaccine-related information. Respondents, especially HCWs, showed a marked preference for institutional and scientific formats, such as training courses accredited for continuing education credits (ECM), webinars, and official websites. This preference suggests that digital health literacy may be leveraged to counteract vaccine hesitancy and misinformation, particularly if integrated into regular institutional training programmes. The potential for digital platforms to enhance adult vaccine uptake has been documented in previous studies [11,21], and should be strategically explored in hospital communication plans.

The cross-sectional study presents several strengths that enhance its scientific value and relevance. The inclusion of a large and diverse sample—comprising participants from both healthcare (physicians, nurses, technicians, auxiliary staff) and administrative sectors—allows for a broad, inclusive analysis of professional groups that are rarely studied together. The focus on pneumococcal vaccination, a topic often overlooked in research on healthcare workers—which typically emphasises influenza or COVID-19 vaccines—adds a unique perspective. The use of robust statistical methods, including multivariable logistic regression across professional categories, strengthens the reliability of the findings and enables the identification of meaningful associations between sociodemographic factors and vaccine-related knowledge or hesitancy. Furthermore, the setting of a large Italian university hospital addresses a notable gap in the national literature and provides original data that can inform locally adapted vaccination strategies and educational interventions.

Nevertheless, the study provides robust evidence on existing knowledge gaps, motivational drivers, and structural barriers to pneumococcal vaccination in the hospital workforce. These insights highlight the need for stratified and role-specific educational interventions, supported by institutional policies that facilitate access and tracking of vaccination status. Furthermore, the statistically significant differences in perception and attitudes among groups emphasise the importance of cultural and professional context in shaping vaccine behaviour—an area deserving further investigation.

Despite these strengths, several limitations of the study must be acknowledged. The single-centre design restricts the generalizability of the findings to other hospital settings or geographic areas; the results should therefore be interpreted as institution-specific and exploratory in nature. Moreover, the cross-sectional design precludes any causal inference regarding determinants of vaccination behaviour; observed associations should be considered hypothesis-generating. The voluntary response sampling approach carries an inherent risk of selection bias: employees who chose to participate may have had stronger pre-existing interest in vaccination or higher health literacy compared to the general workforce, potentially inflating estimates of knowledge and vaccine acceptance. Self-reported data are susceptible to recall bias and social desirability bias, particularly with respect to past vaccination history. Although the questionnaire used was pilot-tested, it lacked formal validation. Self-reported responses also carry the risk of recall bias or social desirability bias, especially regarding past vaccination behaviours. Moreover, the recent introduction of PCV20—which simplifies the vaccination schedule to a single dose—occurred after ethics committee approval of the survey and was therefore not captured by this study; this change may positively influence future adherence and coverage rates [20].

Future research should adopt a multicentric design and longitudinal follow-up to explore whether targeted interventions can sustainably improve vaccine uptake and reduce hesitancy across healthcare settings. Future studies should also consider extending the survey to non-hospital professional settings—including schools, universities, and community organisations—in order to capture a broader spectrum of vaccination attitudes and barriers among the adult working population and to inform vaccination programmes beyond the hospital environment.

Beyond the institutional context, the findings of this study carry broader public health implications. Healthcare workers represent a critical interface between the healthcare system and high-risk patient populations—including older adults and individuals with chronic conditions—who are the primary targets of pneumococcal vaccination programmes. When HCWs are themselves vaccinated and well-informed about pneumococcal disease, they are more likely to proactively recommend vaccination to their patients, thereby amplifying the population-level reach of immunisation programmes beyond what formal public health campaigns can achieve. Conversely, knowledge gaps and hesitancy among HCWs may generate missed opportunities for recommendation and referral, with downstream consequences for vaccination uptake in vulnerable groups. This is particularly relevant in the context of the Italian National Vaccine Prevention Plan 2023–2025, which targets a minimum vaccination coverage of 75% in at-risk populations—a goal that is unlikely to be achieved without active engagement from hospital-based healthcare professionals.

## 5. Conclusions

This study highlights a concerning gap in pneumococcal vaccination coverage among both healthcare and administrative staff within a major Italian university hospital, despite the availability of free vaccines and clear national recommendations. The heterogeneity in vaccine knowledge, perception, and access preferences between professional categories—especially between clinical and non-clinical personnel—underscores the need for targeted interventions.

The findings support the implementation of structured educational programmes, tailored to the specific knowledge needs and communication preferences of different staff categories. Furthermore, institutional efforts to improve on-site vaccine delivery may enhance uptake, particularly among administrative and auxiliary personnel.

To strengthen preventive strategies and protect vulnerable patients, it is essential for healthcare institutions to invest in comprehensive and inclusive vaccination campaigns, leveraging training formats, trust-building communication, and seamless access. Future research should expand to multiple hospital settings to identify broader sociodemographic determinants of hesitancy and evaluate the long-term impact of tailored interventions. Extending this type of investigation to non-hospital settings, such as schools, universities and community workplaces, would be a valuable direction for future research, enabling the development of vaccination strategies with broader population reach.

## Figures and Tables

**Table 1:** Section (A–B): Descriptive analysis of demographic, occupational, and health-related characteristics—HCWs and administrative staff.

Variable		HCWs N (%)	Administrative Staff N (%)	*p*-Value
**Gender**	Male	246 (27.55)	66 (33.51)	
	Female	641 (71.78)	128 (64.97)	0.01
	I prefer not to answer	6 (0.67)	3 (1.52)	
**Age**		M (IQR)	M (IQR)	0.002
		48 (38–56)	48 (38–56)	
**Education**	Middle school	28 (3.13)	6 (3.05)	
	High School	150 (16.80)	98 (49.75)	<0.001
	University	276 (30.91)	51 (25.89)	
	Postgraduate	439 (49.16)	42 (21.31)	
**Marital status**	Married	473 (52.96)	104 (52.79)	
	Cohabiting	101 (11.31)	24 (12.18)	0.33
	Widower	6 (0.67)	5 (2.54)	
	Divorced	70 (7.84)	20 (10.15)	
	Single	225 (25.19)	44 (22.34)	
	I prefer not to answer	18 (2.03)	0	
**Smoking**	Yes	218 (24.42)	47 (23.86)	
	No	675 (75.58)	150 (76.14)	0.09
**Chronic condition**	Yes	254 (28.47)	56 (26.44)	
	No	639 (71.53)	141 (71.56)	0.1

**Table 2:** Section C: Knowledge and attitudes on vaccination in general and pneumococcal vaccination—HCWs.

ITEMS		Medical and Specialist Healthcare Professionals	Nursing, Technical and Rehabilitative Healthcare Professionals	Healthcare Assistant and Auxiliary Technical Staff	*p*-Value
**Do you know how many types of pneumococcal vaccines are available?**	0	7 (2.56)	22 (3.82)	12 (27.27)	<0.001
	1	41 (15.02)	153 (26.56)	7 (15.91)	
	2	132 (48.35)	270 (46.88)	22 (50.00)	
	>2	93 (34.07)	131 (22.74)	3 (6.82)	
**Vaccines are an essential tool for the protection of the individual and the community.**	1. Strongly disagree	4 (1.48)	5 (0.87)	1 (2.27)	<0.001
	2. Disagree	1 (0.37)	14 (2.43)	3 (6.82)	
	3. Agree	36 (13.28)	273 (47.32)	30 (68.18)	
	4. Strongly agree	230 (84.87)	285 (49.38)	10 (22.73)	
**Vaccines have negligible impact on the spread of infectious disease.**	1. Strongly disagree	202 (74.54)	204 (35.36)	7 (15.91)	<0.001
	2. Disagree	42 (15.49)	234 (40.59)	15 (34.09)	
	3. Agree	15 (5.54)	117 (20.28)	19 (43.18)	
	4. Strongly agree	12 (4.43)	22 (3.91)	3 (6.82)	
**The risk of complications from pneumococcal disease is higher than the risk of serious adverse effects from vaccines.**	1. Strongly disagree	16 (5.90)	18 (3.12)	3 (6.82)	<0.001
	2. Disagree	5 (1.85)	78 (13.52)	7 (15.91)	
	3. Agree	76 (28.04)	328 (56.84)	29 (65.91)	
	4. Strongly agree	174 (64.21)	153 (26.52)	5 (11.36)	
**Protecting immunity against Pneumococcus through pneumococcal infection is preferable to obtaining it through vaccination.**	1. Strongly disagree	130 (47.97)	122 (21.14)	2 (4.54)	<0.001
	2. Disagree	113 (41.70)	294 (50.96)	21 (47.73)	
	3. Agree	20 (7.38)	139 (24.09)	21 (47.73)	
	4. Strongly agree	8 (2.95)	22 (3.81)	0 (0.00)	
**Pneumococcal vaccination helps prevent the spread of antibiotic resistance.**	1. Strongly disagree	17 (6.27)	18 (3.12)	1 (2.27)	<0.001
	2. Disagree	10 (3.70)	73 (12.65)	6 (13.64)	
	3. Agree	102 (37.64)	357 (61.87)	33 (75.00)	
	4. Strongly agree	142 (52.39)	129 (22.36)	4 (9.09)	
**Pneumococcal disease is a serious health risk.**	1. Strongly disagree	6 (2.21)	6 (1.04)	2 (4.55)	<0.001
	2. Disagree	6 (2.21)	27 (4.68)	2 (4.55)	
	3. Agree	92 (33.95)	358 (62.04)	34 (77.26)	
	4. Strongly agree	167 (61.63)	186 (32.24)	6 (13.64)	
**Available pneumococcal vaccines are safe.**	1. Strongly disagree	0 (0.00)	3 (0.52)	1 (2.27)	<0.001
	2. Disagree	1 (0.37)	31 (5.37)	5 (11.36)	
	3. Agree	139 (51.29)	441 (76.43)	36 (81.82)	
	4. Strongly agree	131 (48.34)	102 (17.68)	2 (4.55)	
**PCV-13 vaccine offers sufficient level of protection for pneumococcal infections.**	1. Strongly disagree	1 (0.37)	6 (1.04)	1 (2.27)	<0.001
	2. Disagree	24 (8.86)	48 (8.32)	4 (9.09)	
	3. Agree	181 (66.79)	463 (80.24)	39 (88.64)	
	4. Strongly agree	65 (23.98)	60 (10.40)	0 (0.00)	
**Consequential PCV13 + PPSV-23 vaccination is necessary to achieve complete protection from pneumococcal disease.**	1. Strongly disagree	0 (0.0)	7 (1.21)	1 (2.27)	<0.001
	2. Disagree	16 (5.90)	56 (9.71)	7 (15.91)	
	3. Agree	183 (67.53)	449 (77.81)	35 (79.55)	
	4. Strongly agree	72 (26.57)	65 (11.27)	1 (2.27)	
**PCV-13 and PPSV-23 vaccines can cause serious side effects and/or adverse reactions.**	1. Strongly disagree	53 (19.56)	22 (3.81)	2 (4.55)	<0.001
	2. Disagree	141 (52.03)	309 (53.55)	17 (38.64)	
	3. Agree	68 (25.09)	223 (38.65)	24 (54.54)	
	4. Strongly agree	9 (3.32)	23 (3.99)	1 (2.27)	

**Table 3:** Section D: Vaccination-related intentions, behaviours, and attitudes—HCWs.

ITEMS		Medical and Specialist Healthcare Professionals	Nursing, Technical and Rehabilitative Professionals	Healthcare Assistant and Auxiliary Technical Staff	*p*-Value
**Have you ever been vaccinated with the pneumococcal vaccine?**	Yes	65 (24.07)	113 (19.82)	3 (7.14)	0.001
	No	205 (75.93)	457 (80.18)	39 (92.86)	
**On a scale of 1 (not at all) to 4 (very much), how good do you think your knowledge about the Pneumococcal vaccine is?**	1	37 (13.70)	145 (27.31)	17 (40.48)	0.001
	2	135 (50.00)	273 (51.42)	16 (38.10)	
	3	78 (28.89)	91 (17.14)	4 (9.52)	
	4	20 (7.41)	22 (4.14)	5 (11.90)	

**Table 4:** Multivariable regression between medical and specialist healthcare professionals, nursing, technical and rehabilitative professionals and healthcare assistant/auxiliary technical staff (Pseudo R2 0.1649).

ITEMS	Job Category		
	Nursing, Technical and Rehabilitative Professionals	Medical and Specialist Healthcare Professionals OR [IQR] *p*-Value	Healthcare Assistant and Auxiliary Technical Staff OR [IQR] *p*-Value
**Vaccines are an essential tool for the protection of the individual and the community**	BASE OUTCOME	1.71 [1.17–2.48] 0.005	0.71 [0.36–1.38] 0.313
**Vaccines have negligible impact on the spread of infectious disease**		0.64 [0.51–0.80] <0.001	1.43 [0.90–2.29] 0.124
**The risk of complications from pneumococcal disease is higher than the risk of serious adverse effects from vaccines**		1.47 [1.16–1.86] 0.001	0.63 [0.38–1.09] 0.97
**Protecting immunity against Pneumococcus through pneumococcal infection is preferable to obtaining it through vaccination**		0.78 [0.61–0.99] 0.043	1.36 [0.77–2.38] 0.279
**Pneumococcal vaccination helps prevent the spread of antibiotic resistance**		1.09 [0.86–1.39] 0.478	1.20 [0.62–2.33] 0.583
**Pneumococcal disease is a serious health risk**		0.95 [0.69–1.31] 0.772	0.56 [0.30–1.06] 0.201
**Available pneumococcal vaccines are safe**		2.02 [1.32–3.10] 0.001	0.74 [0.26–2.09] 0.570
**PCV-13 vaccine offers sufficient level of protection for pneumococcal infections**		0.86 [0.59–1.26] 0.438	1.86 [0.58–6.04] 0.296
**Consequential PCV13 + PPSV-23 vaccination is necessary to achieve complete protection from pneumococcal disease**		1.26 [0.85–1.87] 0.242	0.76 [0.30–2.00] 0.590
**PCV-13 and PPSV-23 vaccines can cause serious side effects and/or adverse reactions**		0.77 [0.59–1.02] 0.068	0.92 [0.50–1.73] 0.816

**Table 5:** Multivariable regression between healthcare workers and administrative staff (Pseudo R2 0.0285).

ITEMS	Job Category	
	Healthcare Staff	Administrative Staff OR [IQR] *p*-Value
**Vaccines are an essential tool for the protection of the individual and the community**	BASE OUTCOME	0.93 [0.67–1.30] 0.68
**Vaccines have negligible impact on the spread of infectious disease**		1.07 [0.87–1.33] 0.52
**The risk of complications from pneumococcal disease is higher than the risk of serious adverse effects from vaccines**		0.70 [0.56–0.87] 0.002
**The acquisition of immunity against pneumococcus through pneumococcal infection is preferable to that conferred by vaccination**		0.99 [0.78–1.25] 0.945
**Pneumococcal vaccination helps prevent the spread of antibiotic resistance**		1.22 [0.93–1.63] 0.148 0.80 [0.58–1.10]
**Pneumococcal disease poses a serious health risk**		0.178
**Available pneumococcal vaccines are safe**		0.71 [0.48–1.07] 0.178

## Data Availability

The data obtained for this study will not be shared due to “personal data protection”. Collective consent of the participants is required for sharing. However, all data generated or analysed during this study are already included in article.

## Supplementary Materials

The following supporting information can be downloaded at https://www.mdpi.com/article/10.3390/vaccines14060530/s1, Table S1: Section D Vaccination-related intentions, behaviours, and attitudes—HCWs. Table S2: Section E Preferred Channels for Vaccine Information—Administrative Staff. Table S3: Section C Knowledge and attitudes on vaccination in general and pneumococcal vaccination according to educational level—Administrative staff.

## Abbreviations

HCWs	Healthcare workers
PCV	Pneumococcal Conjugate Vaccine
PPV	Pneumococcal Polysaccharide Vaccine
Flu	Influenza
