# Supplementary material for: Knowledge, Attitudes and Perceived Barriers to Pneumococcal Vaccination: A Cross-Sectional Survey Among Healthcare Workers and Administrative Staff at an Italian University Hospital
Source: Vaccines (Basel). 2026 Jun 15;14(6):530. doi: 10.3390/vaccines14060530 (PMC13307725; doi:10.3390/vaccines14060530)
Supplement: Supplementary file 1 [file vaccines-14-00530-s001.zip › vaccines-4328982-supplementary.pdf]

Table S1. Section E Preferred Channels for Vaccine Information. - HCWs

ITEMS		*Medical and specialist healthcare professionals*	*Nursing, technical and rehabilitative professionals*	**Healthcare assistant and auxiliary technical staff**	***p-value***
**Through which channels would you like to gain more knowledge about pneumococcal vaccines?**	Webinars	92 (26.36)	132 (18.59)	5 (12.82)	0.578
	Continuing education credits	116 (33.24)	364 (51.57)	7 (15.95)	
	Institutional websites	114 (32.66)	160 (22.54)	25 (64.10)	
	Podcast	27 (7.74)	54 (7.61)	2 (5.13)	

Table S2. Section E Preferred Channels for Vaccine Information – Administrative Staff.

ITEMS		**Middle school**	**High school**	**University degree**	*Post-graduate*	***p-value***
**Through which channels would you like to gain more knowledge about pneumococcal vaccines?**	In-person events	4 (57.14)	21 (17.65)	9 (12.68)	8 (13.79)	0.892
	Online Events	1 (14.29)	33 (27.73)	23 (32.39)	17 (29.31)	
	Institutional websites	2 (28.57)	57 (47.90)	33 (46.48)	25 (43.10)	
	Podcast	0 (0.00)	8 (6.72)	6 (8.45)	8 (13.79)	

Table S3. Section C Knowledge and attitudes on vaccination in general and pneumococcal vaccination according to educational level - Administrative staff.

ITEM		Middle School	High school	University Degree	Post-graduate	*p - value*
**Do you know how many types of pneumococcal vaccines are available?**	0	2 (33.33)	30 (30.00)	7 (14.00)	5 (12.20)	**0.009**
	1	3 (50.00)	26 (26.00)	21 (42.00)	6 (14.63)	
	2	0 (0.00)	29 (29.00)	12 (24.00)	18 (43.90)	
	>2	1 (16.67)	15 (15.00)	10 (20.00)	12 (29.27)	
**Vaccines are an essential tool for the protection of the individual and the community**	Strongly disagree	0 (0.00)	0 (0.00)	0 (0.00)	0 (0.00)	0.065
	Disagree	0 (0.00)	2 (2.04)	0 (0.00)	1 (2.44)	
	Agree	3 (50.00)	66 (67.35)	28 (56.00)	15 (36.59)	
	Strongly agree	3 (50.00)	30 (30.61)	22 (44.00)	25 (60.97)	
**Vaccines have negligible impact on the spread of infectious disease**	Strongly disagree	2 (33.33)	28 (28.00)	18 (36.00)	22 (53.65)	0.213
	Disagree	3 (50.00)	45 (45.00)	21 (42.00)	12 (29.27)	
	Agree	1 (16.67)	24 (24.00)	8 (16.00)	4 (9.76)	
	Strongly agree	0 (0.00)	3 (3.00)	3 (6.00)	3 (7.32)	
**The risk of complications from pneumococcal disease is higher than the risk of serious adverse effects from vaccines**	Strongly disagree	0 (0.00)	4 (4.00)	3 (6.00)	2 (4.88)	0.134
	Disagree	1 (20.00)	20 (20.00)	10 (20.00)	6 (14.63)	
	Agree	3 (60.00)	62 (62.00)	31 (62.00)	18 (43.90)	
	Strongly agree	1 (20.00)	14 (14.00)	6 (12.00)	15 (36.59)	
**Protecting immunity against Pneumococcus through pneumococcal infection is preferable to obtaining it through vaccination**	Strongly disagree	1 (20.00)	22 (22.00)	14 (28.00)	15 (37.50)	0.187
	Disagree	2 (40.00)	38 (38.00)	27 (54.00)	13 (32.50)	
	Agree	2 (40.00)	34 (34.00)	6 (12.00)	10 (25.00)	
	Strongly agree	0 (0.00)	6 (6.00)	3 (6.00)	2 (5.00)	
**Pneumococcal vaccination helps prevent the spread of antibiotic resistance**	Strongly disagree	0 (0.00)	2 (2.00)	3 (6.00)	3 (7.50)	**0.032**
	Disagree	1 (20.00)	11 (11.00)	1 (2.00)	6 (15.00)	
	Agree	4 (80.00)	66 (66.00)	38 (76.00)	18 (45.00)	
	Strongly agree	0 (0.00)	21 (21.00)	8 (16.00)	13 (32.50)	
**Pneumococcal disease is a serious health risk**	Strongly disagree	0 (0.00)	0 (0.00)	1 (2.00)	0 (0.00)	0.604
	Disagree	0 (0.00)	9 (9.00)	2 (4.00)	3 (7.50)	
	Agree	4 (80.00)	70 (70.00)	34 (68.00)	23 (57.50)	
	Strongly agree	1 (20.00)	21 (21.00)	13 (26.00)	14 (35.00)	
**Available pneumococcal vaccines are safe**	Strongly disagree	0 (0.00)	1 (1.00)	1 (2.00)	0 (0.00)	0.156
	Disagree	1 (20.00)	11 (11.00)	3 (6.00)	3 (7.50)	
	Agree	4 (80.00)	69 (69.00)	39 (78.00)	24 (60.00)	
	Strongly agree	0 (0.00)	19 (19.00)	7 (14.00)	13 (32.50)	
